# Paper vs. practice: How legal and ethical frameworks influence public sector data professionals in the Netherlands

**DOI:** 10.1016/j.patter.2022.100604

**Published:** 2022-10-14

**Authors:** Isabelle Fest, Maranke Wieringa, Ben Wagner

**Affiliations:** 1 Utrecht School of Governance, Utrecht University, 3511ZC Utrecht, the Netherlands; 2 Governing the Digital Society, Utrecht University, Utrecht 3512 BR, the Netherlands; 3 Parell Group, Arnheim 6824 BJ, the Netherlands; 4 Department of Technology, Policy and Management, TU Delft, Delft, the Netherlands; 5 Faculty of Creative Futures, Inholland University of Applied Science, TU Delft, Delft, the Netherlands

**Keywords:** DSML2: Proof-of-concept: Data science output has been formulated, implemented, and tested for one domain/problem

## Abstract

Recent years have seen a massive growth in ethical and legal frameworks to govern data science practices. Yet one of the core questions associated with ethical and legal frameworks is the extent to which they are implemented in practice. A particularly interesting case in this context comes to public officials, for whom higher standards typically exist. We are thus trying to understand how ethical and legal frameworks influence the everyday practices on data and algorithms of public sector data professionals. The following paper looks at two cases: public sector data professionals (1) at municipalities in the Netherlands and (2) at the Netherlands Police. We compare these two cases based on an analytical research framework we develop in this article to help understanding of everyday professional practices. We conclude that there is a wide gap between legal and ethical governance rules and the everyday practices.

## Highlights

• “We all know that [..] we should be accountable etcetera […] yes but how?"
• There is a wide gap between legal and ethical governance rules and everyday practices
• Frameworks are integrated implicitly, subtly influencing decision making
• Mainstreaming literacy, rather than specialization, is a necessary consequence

## The bigger picture

There have been intense debates among the general public and the data science community alike about the right way to do data science. Scandals about the misuse of data and abusive practices around data have led to a slew of new ethical and legal frameworks that attempt to govern data science practices. Yet there is little available research on how these legal and ethical frameworks are implemented in practice.

## Teaser

Recent years have seen a massive growth in ethical and legal frameworks to govern data science practices. This paper looks at two cases in the Netherlands: public sector data professionals at municipalities and the Netherlands Police. The paper concludes that there is a wide gap between legal and ethical governance rules and the everyday practices. Legal and ethical frameworks are integrated implicitly, subtly influencing decision making. In consequence, there is a need to mainstream literacy, rather than specialization.

## Introduction

“We all know that we need to explain an algorithm and we should be accountable etcetera … those lofty concepts are familiar, but the question is: ‘yes but how?’" (Field notes, April 19, 2021)

Over the past few years, there has been a massive growth in ethical and legal frameworks to govern data science practices related to data and algorithms. International organizations like the Council of Europe,^1^^,^^2^ the European Union,^3^ and UNESCO,^4^ but also industry associations like IEEE^5^ and ACM,^6^ have released numerous frameworks on how to respond to the rise of algorithmic systems and artificial intelligence. There is also a broad debate within the data science community on ethics, responsibility, and accountability in data science, including considerations about who should be responsible for “doing” ethics in practice^7^ and which “public” ethical frameworks should be designed for.^8^ There are also broad debates on whether frameworks that only provide for transparency are sufficient,^9^ how to appropriately consider harm,^10^, ^11^, ^12^ and the degree to which ethics are insufficient without a meaningful consideration of law and politics.^13^^,^^14^ We also encountered highly relevant debates in the data science community about the role of discretion in public sector organizations,^15^^,^^16^ with a specific focus on the relationship between algorithms, bureaucracy, and discretion^17^; how discretionary decisions about data influence the outcomes of data science^18^; the link between trust and discretion^19^; and the degree to which discretion can be automated.^20^

Beyond academic debates, there are also numerous novel legal frameworks that attempt to governance everyday data science practices, such as the EU’s General Data Protection Regulation (GDPR) or the upcoming EU Artificial Intelligence Act.^21^ Given this flurry of novel ethical and legal frameworks that all attempt to govern data science practices related to data and algorithms, it is interesting to see to what extent these novel ethical and legal frameworks influence the everyday practices of data professionals. If, as proposed by Caitlin D Wylie,^7^ everyday data science practitioners should be responsible for making ethical decisions, it is important to study what data science practitioners’ ethical decisions look like in practice.

We argue it is insufficient to look “just” at data scientists themselves, but rather it’s necessary to understand the broader context in which data scientists operate. To study both communities in a meaningful way, we use the term “data professionals” to cover both the data scientists directly and the civil servants developing policy for the data scientists. Moreover, in practice, we found that many people working with or on data did not uniformly identify as one particular group such as “data scientist,” “data analyst,” or “data policy advisor.” Rather than reducing our respondents’ nuanced perception of their own position, we opted to use the more all-encompassing term of data professional, which acknowledges the fluidity of their positions.

To study the everyday ethical and legal decisions of data scientists, it is particularly interesting to look at the everyday practices of data professionals working in the public sector. This is because individuals working in the public sector typically operate in an environment where responsibility and accountability are more important than in comparable private sector positions.^22^ Part of the reason for this is due to the increasing shift toward data-centric processes and systems in the area of public government,^23^ within which public servants and algorithmic systems work together.^15^

Within the public sector, this leads to frequent “ethical and legitimacy challenges.”^23^ These challenges around ethics and legitimacy piqued our interest to look more closely at novel ethical and legal frameworks being used. For the purpose of our analysis, we believe it is most interesting to look at an environment where data science practices are common across different parts of the public sector, to be able to contrast them in multiple public sector domains. We decided to take a closer look at the Netherlands, where the usage of data science practices as part of everyday public sector decision making are already relatively normal and well established.^24^ However, it remains unclear to what extent these novel ethical and legal frameworks influence the everyday practices of public sector workers in the Netherlands. This leads to our central research question: How do ethical and legal frameworks influence the everyday practices on data and algorithms of public sector data professionals in The Netherlands?

This article will answer this research question by analyzing two cases in the Netherlands in which data professionals engage in data science practices as part of their everyday public decision making. In conclusion, we will argue that high-level declarations about AI ethics and even legal regulation have little *direct* effect on the everyday practices of professionals working with data and algorithms. However, many of the relevant substantive issues tackled by high-level ethical and legal regulatory proposals are being engaged with in practice.

## Experimental procedures

### Resource availability

#### Lead contact

The lead contact for questions about this is Ben Wagner who can be reached at (ben@benwagner.org).

#### Materials availability

This research project has not generated any materials.

### Research design, case selection, and methodology

In order to answer our exploratory research question, we opted for a qualitative interpretative research design, focusing on the perceptions of data professionals at each of our research sites. In contrast to more positivist designs, interpretive research focuses on particular contexts and situated meaning-making processes. By multiple iterations of going back and forth between theory and empirical data, we hoped to unravel particular situated meanings, rather than making generalizable statements as one might expect in positivist research designs.^25^^,^^26^ This qualitative approach allows us to better understand the practices and perspectives of the actors involved.^27^^,^^28^

#### Case selection

As discussed in the introduction, we decided to take a closer look at the Netherlands. The Netherlands have been chosen as a “most advanced practice.” Public sector organizations are actively investing in the development and implementation of algorithmic systems.^23^ As such, the usage of data science practices as part of everyday public sector decision making has been largely normalized. We would expect frameworks to be implemented and discussed more in such a context than contexts where algorithmic systems are less integrated in the public sector. This makes the Netherlands a particularly interesting context to study.

In selecting our cases, we decided to use a most different design,^29^ to ensure that our selection would be "broadly representative of the population will provide the strongest basis for generalization.”^29^ In terms of divergence between and coverage of cases, we considered the different levels of technical knowledge within public sector organizations as well as their varying mandates. After considering different public sector organizations, we settled on studying municipalities in The Netherlands and at the Netherlands Police.

In the Netherlands, municipalities are third-tier local governments, after the national government and the 12 provinces. Due to the decentralized unitary nature of Dutch public administration, many tasks are delegated to the 352 municipalities by the central government, and each municipality has a measure of independence. They are responsible for delivering various kinds of public services from registering citizens in the governmental databases to ensuring welfare benefits to jobless citizens, accommodation and care for disabled persons, realizing infrastructure within their territory, and so forth. A Dutch municipality is divided into two branches: the legislative branch and an executive branch. The legislative branch is composed of the municipal (or respectively, “city”/“island”) council, composed of elected political representatives. Elections are held every 4 years. The executive branch is divided into the executive board and the civil service. Within the group of municipalities there are stark differences, as funding, by and large, is related to the numbers of inhabitants.

As this is an exploratory study and little is yet known on how Dutch municipalities relate to algorithmization,^30^ we chose to cast the net wide across Dutch municipalities rather than immediate focus on specific municipalities that may show different kinds of interactions with legal frameworks. Our data cover 25 specific municipalities ranging from some municipalities that have devoted a large number of resources to the data-driven transformation and small municipalities that have barely begun that transition.

The Netherlands Police is the national police service with a mandate of law enforcement. In 2013, the Netherlands police was established as a centralized police force, which replaced a total of 25 regional and one national corps, which had all functioned autonomously. As a result of this reorganization there has been an increasing influence from the central level, while much regional autonomy remains. Many formal and informal networks around algorithmic systems have since emerged, for example on a project level, within local or regional departments, or between data professionals across departments.

The Netherlands Police develop the majority of the algorithmic technologies they use themselves and as such have much more in-house technical knowledge than municipalities in The Netherlands, which tend to buy technical systems from contractors due to limited operational capacity. At the same time, the mandates of the two organizations are very different, with municipalities having a political-administrative mandate and the police having considerable legal and regulatory burden due to their public policing mandate. As such municipalities in the Netherlands and the Netherlands Police should be considered most different cases.

#### Method

Algorithmic systems are often considered opaque or even referred to as black boxes—difficult if not impossible to understand and analyze or scrutinize. This is further strengthened by the sensitivity of algorithmic and data projects in the Netherlands, which can be related to recent public scandals that received much media attention. Particularly the SyRI scandal and the Child Benefits Scandal have placed public sector organizations on higher alert when it comes to implementation of algorithmic systems, making access more difficult to negotiate in some cases.^31^ In dealing with this, we employed the ethnographic practice of “scavenging” for material. As Seaver notes, “A great deal of information about algorithmic systems is available to the critic who does not define her object of interest as that which is off limits or intentionally hidden."^32^ This is particularly true for our research, as we are interested in the organizational practices surrounding algorithmic design rather than their code.

We thus scavenged our data, gathering it sometimes through direct observation of participants’ work practice or meetings and at other times through court hearings or public documents over a 4-year time frame between 2017 and 2021. We rely on semi-structured interviews, observations of daily practice, and informal conversations and interactions by the coffee machine. Some of this scavenged material resulted in field notes (municipalities: 94, police: 3) and interview transcripts (municipalities: 3, police: 21), which form the core data for the current paper (see Table 1). Other scavenged material has played a more invisible role in shaping and adapting our research design and thinking. As is common with ethnographic methodologies and interpretive research designs, not all the work that has been put into it can be represented in a quantitative manner.

**Table 1:** Scavenged data

	Time frame	Semi-structured interviews	Field notes	Amount of organisations covered
Municipalities	November 2018 – June 2021	3	94	25
Police	September 2020 – April 2022	21	3	1

For Dutch municipalities, we conducted a total of three semi-structured interviews (of approximately 45 min) and had 93 interactions with and/or observations of data professionals in this field. Some of these interactions lasted a day or more; in other cases, the interactions were shorter. In some instances, we had the opportunity to visit a particular project group or individual several times, but more often we sat in on (semi-)public inter-municipality meetings, had conversations with civil servants about their organization, were invited to data awareness events in which we would give a presentation and would otherwise observe, and so forth. Our municipal investigations concern a wide overview of the overall Dutch state of how frameworks are used within municipalities. Our municipal data professionals ranged from data scientists to privacy officers and from policy advisors to data ethics experts. This is a rather heterogeneous group, but they all are in some way or another working with/on data and algorithm use, implementation, and/or development, making them an interesting group to study in translating the abstract frameworks to practice.

With the Netherlands Police, we conducted a total of 21 1-h qualitative semi-structured interviews with data professionals, as well as observations of a total of three online meetings of the “Data Science Community” (DSC) at the Netherlands Police. This comprised roughly 5 h of observation. During these meetings, which are organized once every 2 months, data scientists and other interested parties from across the police organization discuss data science projects and related matters. Meetings include several presentations of (recent) work or projects, followed by a discussion. Most participants of these meetings have a technical background, which was reflected in the discussions. Although the core of these meetings was technical in nature, other rules, regulations, and ethical considerations were always implicitly present and often explicitly mentioned.

For both research sites, the researchers were granted approval to conduct interviews by the Ethical Committee of their respective institutions (municipalities: FETC-GW 5670705-01-01-2020; police: FETC-REBO “Value-sensitive algorithmization in the Netherlands Police”). Interviews were transcribed verbatim, and interviewees were assigned an identification number (municipalities) or pseudonym (police). When observing or interacting with participants outside of interviews, researchers clearly stated their affiliation and research interest. For each of the research sites, our raw data were uploaded to NVivo for multiple consecutive rounds of open, axial, and selective coding.^33^ In line with interpretative research methods, we employed an abductive strategy in coding, going through various iterations of (1) searching for wider patterns across our data, (2) finding empirical evidence for these patterns at the sentence level, and (3) connecting patterns and evidence to theory. Findings were discussed among the researchers and compared by including raw data from both research sites in a final round of selective coding using the framework developed in the previous section as a starting point.

Timelines between the research sites varied due to practical considerations. Our observations within municipalities began in the aftermath of the GDPR implementation, whereas we started our investigation in the Netherlands Police in 2020, during a pandemic. We thus observed not only the changes to legislation and policy in this field over the course of several years but also how these civil servants to some degree had to reorient their work practice in light of COVID-19.

### Analytical research framework

We were particularly interested to understand how ethical and legal frameworks as types of professional rules and standards are implemented in practice.^34^ The way in which practitioners implement these frameworks is particularly interesting, as they do not have specific ethical or legal knowledge per se. In this sense, our research follows the proposal by Caitlin D. Wylie “that the responsibility for serving society through good data work lies with practitioners—all of them.”^7^ We will study what happens when data professionals take on this responsibility using ethical and legal frameworks. The everyday practices of data scientists are of course not just influenced by professional rules and standards or ethical considerations but by numerous other factors as well. For example, data scientists are typically influenced by the data governance strategies, which in turn influences the interpretation of data.^35^ There are also considerable concerns about the degree to which ethical and legal frameworks that provide greater transparency are able to provide meaningful accountability.^9^ It has even been argued that the concept of ethics is insufficient and that data science must instead embrace their role as political actors.^13^ While we support Green’s assertion about the political nature of data science that goes beyond what can be captured in ethical frameworks, the current flurry of novel ethical and legal guidelines is an empirical phenomenon in and of itself that is worth further academic study.

In order to meaningfully interpret data professionals' practices that we observe and relate them to the research question, we have developed an analytical research framework to structure the collected data. We use the analytical research framework to structure and analyze the two cases. Additionally, as we are working with the framework in practice, we will critically engage with it as researchers. Through this, we hope to generate a deeper understanding of how frameworks “work” in practice, and thus how data professionals in municipalities and police may engage with such frameworks. Moreover, we believe these two cases are interesting because they engage with how everyday practices are shaped at two different levels. We look at data professionals who are responsible for developing and drafting the actual practices around data as well as those who implement these into everyday data science practices.

The purpose of the analytical research framework is explicitly not to create a novel or even superior set of ethical principle that we expect practitioners to follow or will measure practitioners’ performance against. Rather it is a useful structuring device to ensure that we are looking at all relevant ethical areas that are discussed in the academic literature. As such we do not claim that our framework is better or worse than any other framework but rather that it should be considered as a useful mapping of relevant issues. In doing so we also draw on existing literature from the data science community, in particular in areas such as thinking about harm.^10^, ^11^, ^12^

Our analytical research framework is based on ethical frameworks for data scientists developed by a public sector organization working on this topic in the UK Cabinet office,^36^ which defines the six main principles for data scientists in the UK. We also build on a literature review of ethical considerations in data science by Saltz and Dewar,^37^ which documents seven main ethical areas of focus. By drawing on both Drew as well as Saltz and Dewar in our own combined research framework, we believe it is possible to address the needs of both the public sector and ethical frameworks for data professionals’ practices more broadly.

When applying our research framework, we will systematically integrate our central research question on the role of ethical and legal frameworks. Our combined analytical research framework is based on these two existing frameworks, while integrating other key aspects from other ethical and legal frameworks that exist at an intergovernmental level such as the EU’s Ethics Guidelines for Trustworthy AI.^1^^,^^3^^,^^38^^,^^39^

It is important to consider here that the boundaries between legal and ethical frameworks are blurring considerably around algorithms and AI, with both presented as rule-based frameworks that may be binding on actors. While legal frameworks tend to be based on constitutional principles, ethical frameworks also often overlap with them in areas such as non-discrimination. However ethical frameworks are often seen as more flexible and thus often preferred as a form of “light-touch” governance over more strict legal regulation.^14^ However, the flexibility of ethical frameworks often also means definitions are broad and vague, lacking sufficient legal certainty to ensure effective implementation. Crucially, many proposed ethical frameworks around algorithms and AI are quite far from scholarly understandings of what “doing ethics” should be^7^^,^^10^ or professional ethical conduct.^40^

This result of the synthesis in Table 2 is our own analytical research framework that we used as a research tool to structure and categorize the empirical data we are collecting. This framework is particularly valuable as foundational categories to structure our comparison below (see Comparison).

**Table 2:** Synthesis of key ethical frameworks for data science to develop our own analytical research framework

Drew^36^	Saltz and Dewar^37^	EU HLG Ethics guidelines for trustworthy AI^3^	Our analytical research framework
Start with clear user need and public benefit.	–	–	*Mission:* Start with clear user need and public benefit.
–	–	“lawful, complying with all applicable laws and regulation” (p.2)	*Lawful basis:* ensure that you have a lawful basis for your work and that it follows all relevant legal frameworks.
–	–	“fundamental rights-based approach“ (p.5)	*Human Rights and Good Governance:* ensure that your work enables and promotes human rights and good governance.
Use data and tools that have the minimum intrusion necessary.	Data privacy & anonymity	–	*Privacy and Security:* Use data and tools that have the minimum intrusion necessary to safeguard privacy and anonymity, while keeping data secure.
Keep data secure.		–	
Create robust data science models.	Subjective model design	–	*Subjective models:* Create robust data science models that acknowledge their subjectivity and inbuilt assumptions.
	Data misuse	–	
Be alert to public perceptions.	–	–	*Clear Communication:* Be alert to public perceptions and the ways in which your models and results will be interpreted by decision makers and the general public.
Be as open and accountable as possible.	Team accountability	–	*Accountability and Responsibility:* Be as open and accountable as possible, in particular by implementing (external) auditing mechanisms.
–	Personal and group harm	–	**Harm**: Be aware of and attempt to prevent any personal and group harm and take steps to avoid these harms.
–	Misuse/misinterpretation	–	**Misuse**: Ensure that any misuse of results and associated data is prevented.
–	Data accuracy	–	**Accuracy**: Ensure accuracy of results and clearly communicate margins of error of models and data to avoid misinterpretation or misuse.

### Cases

As previously noted, we analyze and compare data from data professionals at (1) municipalities and (2) police in the Netherlands. As both research sites share some overarching laws, rules, and frameworks, we will first introduce these before delving into the two sites in depth. Operating within the Netherlands, Dutch laws and regulations are an obvious common point of reference, as are European laws and regulation. The most prominent legal frameworks are the Dutch constitution, the General Administrative Law Act (Awb), the General Principles of Good Governance (ABBB), the Dutch guidelines for using algorithms by public authorities, and the EU General Data Protection Regulation (GDPR). As the Dutch State is party in numerous human rights conventions, these would apply to both cases as well. One example hereof is the European Convention of Human Rights (ECHR), but also the EU Charter of Fundamental Rights. The remainder of this section will shortly introduce both cases and the laws, regulations, and frameworks that apply to them specifically.

#### Dutch municipalities

Our first research site concerns Dutch municipalities. Due to the decentralized nature of the Netherlands, municipalities have considerable autonomy. As noted, municipalities need to operate within Dutch and EU laws and regulations, but they have an extent of freedom of how to position themselves within these limitations. In line with this, the executive branch of the Association of Dutch Municipalities drafted the Principles for the Digital Society,^41^ in which a common ambition and framework for dealing with public value and digitalization is formulated, but which also leaves room for local interpretations of these principles. Moreover, some municipalities have formulated ethical guidelines for themselves or in collaboration with others. The municipality of Amsterdam, for instance, co-authored the TADA^42^ principles, and the municipality of Nijmegen wrote their manifest called “Open and Resilient.”^43^ Moreover, many municipalities have created/are drafting data strategies, data visions that serve as organizational guidelines on data and algorithm usage.^44^^,^^45^

#### The national police

Our second research site is that of the Netherlands police. Within the Dutch judicial landscape, the national police holds a special position. The General Administrative Law Act (Awb) does not apply to all police tasks and operations, and for those situations, other laws exist. Most notable are The Police Law 2012, which describes the organization of the Netherlands police as well as its core tasks and mandate, and the Police Data Act, which establishes additional regulation about police data use. In addition to the national regulation, the Netherlands Police has also implemented specific policy and frameworks that govern its use of algorithms. The most notable of these is the “Big Data Quality Framework” (Dutch: *Kwaliteitskader Big Data*). This framework is a tool for the assessment of big-data-related projects and guides a project team past a variety of questions concerning legal basis, data quality, fairness issues, etc. This framework is relatively new and has recently begun gaining more traction in the police organization.

### Comparison

Below we will compare both research sites through the research framework we formulated earlier in this paper. For readability’s sake, we have grouped some aspects of the framework and discuss them in a slightly different order to avoid repetition. Where quotes are used, these are all translated from the Dutch by the authors.

#### Mission

The mission denotes whether there is a clear user need and public benefit at the start of the project. For both the municipal and the police context, we found that on a high-level data professionals and civil servants see the creation of public benefit as one of their core tasks (e.g., field notes, October 1, 2019). In both sites, our respondents really feel that this is their *raison d’être* as civil servants and strongly connects to their motivation to work in the public sector. As a municipal data protection officer notes: “In any case we should do what is in the public interest” (T1). However, in both sites we also see struggles in making this concrete in specific projects. For municipalities we find that, despite this overarching mission to create public benefit, individual projects may be framed differently. In some cases, data professionals note that a given project may simply be a pragmatic cost reduction departing predominantly from user need rather than public benefit. Cost reduction, however, is then also framed as a public benefit as the municipality is funded with public funds: “The goal is a better result with less funds” (field notes, October 29, 2020).

For the police the mission is mostly left implicit. While occasionally, it does happen that there is a direct cause for a project to start, with user need and public benefit explicitly known and articulated prior to the project, such projects are exceptions. Most projects are not thought out thoroughly or structurally at the start of each project, and there is usually no explicit notion of user need and public benefit at the start. As noted by one participant, the police mostly practices “undirected innovation” (“Eric”). User need, however, does become very prominent in later stages of the project. Most data professionals report that user experience and satisfaction are the most important factors to determine success, and when in doubt, it is often the users’ opinions that weigh most. As one participant working on an annotation tool explains: “Sure I can build something that works very well on academic data, but if it isn’t used within the police, or if it doesn’t land in the organization. That would be a shame” (“Harmen”). At both research sites we found that efficiency and efficacy are strong drivers for the implementation of algorithmic systems. When it comes to the evaluation of the algorithmic project, efficiency and efficacy are also considered important indicators of success, although they are often difficult to measure.

#### Lawful basis

Both municipalities and the Netherlands Police have a clear lawful basis, as was already described in Cases. Data professionals in both sites are generally aware of the relevant rules and regulations, particularly when it comes to privacy and security. This can be related to the relatively recent implementation of the GDPR, other laws are less often explicitly mentioned. Both organizations also have data protection officers, privacy officers, and legal experts that data professionals consult; however, these seem to play a larger role in municipalities, where we frequently encountered them in meetings, than in our police research site. In the police this is mostly voluntary; experts are regarded as a resource and can be consulted when the data professional has a question or dilemma.

Data professionals at both sites comment on the “gray areas” in legislation; the law does not provide a clear answer in all situations a data professional might deal with in their daily work. Laws, for example, do not mention specific technologies and often lag behind the reality of technological innovation. This makes it difficult for data professionals to translate legislation to their daily practices. We find that data professionals at both municipalities and the police use various tactics to deal with these gray areas. One common method is involving other people. A data professional might ask questions or discuss issues with experts or peers. In the case of the police, a data professional might ask the Public Prosecution Service to make the final call.

A second way to cope with the gray area is the “want to, allowed to, can” test, which is used by municipalities to first assess what they *want* to do, then check what they *are allowed* to do, and then assess what they *can* do in practice.^46^ In the police, this test is not used, and indeed sometimes a data professional might decide to first see whether the technology will work before deciding to ask permission. As one police data professional explains, “We don’t let ourselves get distracted by rules and regulation and really try to experiment first. If things turn out to be useful, and we think we can do something with it, then we sit down with the Public Prosecution Service and lawyers to discuss things like are we even allowed to do this, and how can we make sure it may be used? And that it is juridically sound?” (“Danny”).

#### Human rights and good governance

Our framework pays particular attention to human rights and good governance. In practice, we found that the concept of human rights functions like an umbrella term, and there was a lot of overlap with other points of the framework. Each of the other points of our framework can be linked to human rights, often directly and sometimes indirectly. To avoid duplication, we will limit our discussion in this section to additional points not mentioned elsewhere. Human rights are extensively discussed under other points in this framework, as they are considered increasingly important in both municipalities and the Netherlands police. In consequence, we will not discuss this point further here.

One important aspect of good governance concerns governments’ relationship with citizens. We see a significant difference between our two research sites. Whereas municipalities are particularly close to their citizens, data professionals at the Netherlands Police operate at a greater distance from citizens. As a result, we find municipalities place great importance on citizen participation (for example, round tables with citizens), inclusivity, autonomy, and dignity (for example, emphasis on things like non-discrimination in the design). They often work with impact assessments to implement autonomy and dignity, but implementing participation in practice remains a struggle. One respondent, an advisor of digital ethics in a municipality, notes that “participation is still a … a bit of a gap. (…) We talk about it a lot, but it is talked about like ‘oh yeah, oh yeah, how do we do this?’” (T2).

In contrast, most police algorithms do not *directly* impact citizens and are meant to be used by other police employees. Examples are predictive policing technologies, used to enhance police deployments, or tools that help to sift and search through large amounts of phone data. In such cases, these values play a smaller and more implicit role. One notable exception is the intelligent crime reporting tool (Dutch: *Keuzehulp aangifte*), a chatbot that advises citizens whether to report a case of internet fraud. Despite the challenges with evaluating the long-term impact of such a tool, the police have shown support for (external) research and evaluation and have improved this system based on research. This might indicate a certain degree of responsiveness to the perspectives of citizens.

#### Privacy and security

In both municipalities and the police, privacy and security are important values. They are often discussed and thoroughly implemented. Data professionals can contact privacy or information security officers, there are extensive screening procedures for police employees, and there are technological systems in place that help ensure data security. There is a major difference in the role of data professionals when discussing and implementing these values: for municipalities, they fall under the domain of expert privacy officers and information security officers, and data professionals are usually not directly involved.

For the police, on the other hand, there is generally a high level of awareness of privacy and security, especially among data professionals. They might only contact experts when they have pressing questions. However, one weak spot can be identified when it comes to sharing (citizen) data amongst police employees. As one participant in our police study notes “It surprises me sometimes how easily people send me certain information when I ask for it. When I ask for a specific part, I might suddenly get a list with all kinds of information I did not ask for and which is quite sensitive” (“Laura”). Thus, as some of the data professionals we talked to experience real hurdles accessing data they are entitled to, and some were unwilling to share more specific information in interviews.

#### Subjective models

We find that the amount of significance or attention data professionals attribute to algorithmic limitations depends on the comfortability with data science as a technical discipline. For the Netherlands Police case, we talked mostly to technically oriented data professionals with data science or IT backgrounds. As such, building subjective models and communicating limitations is something participants are very used to. It is core to their work, and they think about such issues on a daily basis. We find a great amount of variation between municipalities. Whereas some have a dedicated data science department and are actively communicating about limitations and subjectivity of models, in other cases data analysis is something civil servants need to do on top of their traditional tasks and with very few people and full-time equivalents (FTEs). As a data manager from a small municipality noted: “A municipality like Amsterdam has an entire data science department, we have to do it on top of what we’re already doing!” (field notes, October 14, 2019). In this case, municipalities might rely on procurement instead of building their own algorithmic models (field notes, October 14, 2019). This can lead to misunderstandings about the limitations and subjectivity of models. We found that communication about subjective models, also overlapped with point 6 of our framework. When communicating algorithmic limitations, data professionals are faced with the challenges mentioned below under internal communication.

#### Clear communication

For communication, respondents from both cases note that there is big difference between internal and external communication.

##### Internal communication and literacy

Internal communication denotes the communication between the data professional and others in the organization, such as decision makers, end users, and other colleagues. Data professionals feel responsible to correctly inform decision makers, policy makers, end users, other civil servants, and in the case of the municipalities, also the municipal council. Data professionals often try to communicate to their non-technical colleagues that their systems are inherently not flawless. One example of this was the need for retraining a police video-analysis algorithm when people started wearing facemasks due to COVID. Data professionals focus on the need for users to make decisions independent of the system, initiate “the good conversation” (Dutch: *Het goede gesprek*), which denotes a structured, cyclical, continuous discussion between in this case project members (T3). They even implement technical solutions to enforce this, such as disclaimers or not highlighting what was found in an image. However, both research sites face a large problem of illiteracy, which complicates such communication. According to one data professional in the police, "(…) there is a pretty big language barrier between the data scientists and the problem owner that wants something solved. They [the problem owners] have a very limited view of what machine learning is. And well that easily leads to Babel-like confusion” (“Aadriaan”).

While knowledge about algorithmic systems is usually present in both municipalities and the police, it tends to be concentrated within specific groups. There is a knowledge gap between the data professionals on the one hand versus end users, managers, policy advisors, and the like on the other. Whereas data professionals have vast insight in technical capabilities and implications of a technology, end users, managers, policy advisors, and the like often have limited knowledge. This impacts their ability to play a role in decision making and places a lot of responsibility and autonomy with the data professionals themselves. Municipalities are investing in knowledge and literacy amongst their civil servants. However, there are stark discrepancies between municipalities. Whereas some municipalities heavily invest in an organization-wide knowledge base and literacy, others barely put data/algorithms on the agenda.

##### External communication

External communication denotes communication to the general public, e.g., communication to citizens or news media. Such communication falls mostly outside the scope of those data professionals we have talked to within the police. For data professionals within the municipalities, this is a very urgent and much discussed topic. For municipalities, external communication is felt to be relatively sensitive due to recent public scandals and upheaval surrounding algorithm use by government organizations in the Netherlands.^31^^,^^47^ Algorithms are considered to be a somewhat volatile topic for many municipalities, and civil servants and data professionals are quite aware of the need for clear communication, with regards to the general public.

Closely related to this concept is transparency, which is sometimes taken by data professionals to be a form of communication to, amongst others, citizens (e.g., field notes, June 4, 2020.), and is gaining importance at both research sites. Data professionals at both research sites are beginning to experiment with and implement explainable AI (XAI) toolkits, which make (technical) decisions more explicit and allows a data professional to communicate their design process. However, such toolkits are very new and not yet widely used (e.g., field notes, January 11, 2022). Another avenue that is currently being explored to increase transparency and communication is algorithm registries, something the House of Representatives in the Netherlands recently voted to make mandatory for governmental organizations. It should be noted that transparency is also very closely related to accountability and responsibility. As explained by one data professional who was given the assignment to create an algorithm registry for the police, the goal of such a registry is “(…) none other than to account for how we obtain our information” (field notes, March 15, 2022).

#### Accountability and responsibility

Data professionals at both municipalities and the police report a high level of felt responsibility for their delivered work as well as the way in which it is used. Our data collected at the Netherlands police do not thoroughly discuss accountability, as this mostly falls outside the domain of those data professionals we talked to. We thus cannot identify any clear patterns based on our data. Within municipalities, data professionals frequently express the lack of “control instruments” for algorithmic systems (e.g., T2; field notes June 4, 2020.). To this end, they are currently developing many tools themselves to account for these systems., the goal of which, a policy advisor from the Association of Dutch municipalities notes, “is to gain more traction on algorithms” (T3). Examples are the drafting of procurement guidelines, drafting methodologies to identify bias and unfairness that can then be communicated and accounted for, the aforementioned algorithm registries, and working to identify whether existing objection procedures are sufficient in light of algorithmic systems. Auditing is increasingly seen across municipalities in general as a way to increase the accountability of and for algorithmic systems. Despite our limited data, we see a similar tendency at the Netherlands police, as some individuals and teams implement accountability measures such as code reviews, where pairs of data professionals look at and improve upon each other’s code (e.g., “Lars”). Please note that there is a large amount of variation within each of our cases. Some municipalities or police teams are more actively discussing accountability and implementing such measures than others.

#### Harm

This touches upon several of the earlier points in this framework. Most notable are the discussions about privacy, human rights, and accountability. As such, we will not repeat the points made in these sections here. As both municipalities and police are regularly under scrutiny when it comes to issues like bias, non-discrimination, and ethnic profiling, preventing harm is considered very important by data professionals at both research sites. Many municipalities are actively designing ways to mitigate bias and prevent discrimination. They often use tools such as Data Ethics Decision Aid (DEDA), Impact Assessment for Algorithms and Human Rights (IAMA), or other impact assessments to identify human rights conflicts and consider and implement appropriate mitigations to limit intrusions (field notes, February 10, 2020; February 13, 2020; May 28, 2020; T3).

We found that a distinction is made between more complex algorithmic systems involving for example neural networks or machine learning on the one hand and more simple algorithmic systems such as administrative tools, regression models, and forms on the other. The term “algorithm” is often used to refer only to those complex systems, particularly by those people in the municipality or police that are less literate when it comes to algorithmic systems. As illustrated by one municipal data professional, the term algorithm may scare people: “If I would say ‘oh, I am participating in a research on algorithms’ then [my colleagues would say] ‘Algorithm? Algorithms?! We don’t use algorithm in [our municipality]?!” Both municipalities and police thus run the risk of focusing on preventing harm in complex systems and overlooking the simpler algorithmic systems in their respective organizations.

#### Misuse

Data professionals in both municipalities and the police are quite mindful of potential misuse of data and algorithmic systems. This includes misuses due to malicious intent or for political gain. In some cases, misuse is prevented through specific choices in the system design process. A data team from a small municipality noted, for instance, that they were asked to create a dashboard for a particular disadvantaged neighborhood and opted instead to create a similar dashboard for the entire municipality, as they felt the data would otherwise miss context and might be leveraged in possibly unsound ways by the city councilors for political reasons (field notes, April 3, 2019.). We find similar examples in several other municipalities as well as the police. Education also plays a role in preventing misuse. For many tools that are ready to be implemented in the organization, users are trained in using the system correctly. Additionally, manuals are written that can help users navigate the systems and prevent accidental misuse.

#### Accuracy

Algorithmic systems can never be 100% accurate; this is an impossibility in data science. As such, in both research sites decisions have to be made about what acceptable levels of accuracy, false positives, and false negatives are. We find data professionals to have a lot of discretion in deciding the acceptable levels of accuracy, and these are (re)determined contextually in each situation. As one data professional in the police explains, “Those are the kind of things data scientists generally just kind of make up” (“Thijmen”). They usually do not have any standards, rules, or regulations to fall back on when making such seemingly technical decisions. Data professionals at both sites thus create different ways of dealing with this. For example, data professionals in one municipality jokingly refer to what they call the “Lisa standard,” which is named after their supervisor. Something is considered good enough if the supervisor agrees with and understands the course of action. (The name “Lisa” is fictitious; field notes, February 9, 2022.) Alternatively, data professionals often take end-user satisfaction to be a measure for acceptability of a system.

### Analysis

#### General attitudes toward frameworks

Overall, we found that data professionals in both municipalities and the police are generally aware of existing rules and regulations – including e.g., laws, frameworks, and relevant ethical guidelines. When it comes to legislation, most data professionals have a positive attitude and feel it is very important such regulation exists. As discussed in Lawful basis, data professionals at both research sites may become frustrated where laws are found lacking. When it comes to other types of frameworks, such as the police’s Big Data Quality Framework or the EU guidelines, participants have more mixed feelings. Some data professionals regard such documents as a very important first step toward more ethical or responsible algorithmization in their organizations, while others feel like they are unnecessary. As one police data professional explains, he prefers rules to be established in laws, and such laws and the professional integrity of employees should be sufficient. “If you have a scientific education, you walk through that entire checklist before writing even one line of code” (“Eric”).

While rules and regulations might be introduced to ensure and safeguard responsible implementation of algorithmic systems, they can be constraining as well. Within both municipalities and the police, data professionals are sometimes frustrated by the limitations such regulation brings to their work. As one participant reflects on the European Commission decision not to use biometric data: “But at the same time, you see this technology advances very quickly. So, it is much easier to recognize persons on camera images. And we *have* cameras, the entire city is full of cameras. So, I sometimes wonder if we are not unnecessarily complicating things for ourselves? But then I think, well, it’s a democracy, it’s not my call. It’s the people’s call, and if we do not want this, we don’t want this so that’s that” (“Lars”).

In conclusion, data professionals are generally aware that rules and regulations governing data science exist. Most data professionals in both municipalities and the Netherlands police have a positive attitude toward such rules and feel it is important such regulation exists. In some cases, they feel more legislation is necessary, particularly when it comes to gray areas currently not covered by laws. There is a distinction to be made between those rules anchored in laws versus those that are not. For some data professionals this creates a confusing situation, where it becomes unclear which rules they need to follow. Although most data professionals are positive about the existence of rules, some data professionals do feel like these rules overly constrain them. In these cases, however, the data professionals tend to accept the constraints around them. In sum, data professionals tend to feel the responsibility of doing “good data work”^7^ and in some cases advocate for more extensive legislation or jurisprudence to mitigate the gray areas (cf. Green^13^; Wagner^14^).

#### Translation to practice

Despite this general positive attitude, we find that data professionals in both municipalities and the police struggle with the practical implementation of these “lofty principles and guidelines” (T3). Translating this in practice remains difficult, they frequently lament (e.g., field notes, January 25, 2022; T2; T3). One of our municipality respondents summed this up in a meeting about a new impact assessment for algorithms and human rights: “A legal expert says ‘we need to adhere to this principle’, a data scientist says ‘so do I need to go left or right?’” (field notes, April 19, 2020.). In an interview, a legal advisor noted something similar: “We all know that we need to explain an algorithm and we should be accountable etcetera … those lofty concepts are familiar, but the question is: ‘yes but how?’" (field notes, April 19, 2021).

We notice the answer to this question in part depends on whether rules are anchored in official laws or not. Laws allow municipalities and police to implement rules through official procedures and approval structures. As these are not optional, motivation to ensure the implementation of such laws is high across all layers of an organization. As a municipal data protection officer notes: “If there is no lawful basis, then the data processing is illegitimate. Full stop! Period!” (T1). Laws may be implemented in various ways, including employee screening procedures, approval of higher authorities, or the involvement of experts such as privacy officers. In municipalities, for instance, intimate knowledge of frameworks tends to be limited to a select number of data professionals (e.g., privacy officer, CISO) and is not necessarily widely shared with the wider project team.

However, those rules anchored in laws concern only a small portion of data professionals’ daily work. As one data professional at the police explains, “(…), the law will never state yes you may do WiFi, but not Bluetooth, or something like that” (T1). There is thus a large gap between those rules we find on paper, for example in laws, frameworks, and ethical guidelines, and the types of decisions or situations a data professional faces in their daily work. At both research sites, it is noted that legal and ethical frameworks are simply not concrete enough for data professionals. (e.g., “Noud”; field notes, April 19, 2021.) The translation from paper to practice appears particularly complex when it comes to those rules not clearly anchored in laws.

As a result, most data professionals we talked to within the police and municipalities report that they do not use frameworks on a regular basis in their daily work. In those few cases where a data professional does regularly work with laws or ethical guidelines, they state that can be fully attributed to a specific project they are working on. As one participant working on an explainable AI project at the police confesses, “before I started this project, I was definitely aware of ethical guidelines, but not how they are explicitly written down. (…) I know it now, but that is due to the project I am working on” (“Lisa”). Implementation of rules and regulation is thus left implicit or falls beyond the data professionals’ perceived responsibility. As noted by one police participant, “in my work, I don’t really bother with the laws and legislature (…). I assume when people want to use these kinds of techniques, that they make sure to satisfy the legal preconditions, and that it is permitted, before they come to me” (“Harmen”).

In conclusion, data professionals struggle with the translation of ethical and legal frameworks to their daily practice. These frameworks tend to be abstract, and interpreting whether a data professional is allowed to do X or Y with technique Z can be difficult. In short, the frameworks are not concrete enough for their daily practice. As a result, the ethical and legal guidelines function differently in practice than envisioned in their creation and implementation (cf. Kuiper^34^).

#### Coping strategies

In an effort to give practical expression to those “lofty” principles we find in rules, both municipalities and the Netherlands police often turn to practical tools. Both organizations use Data Protection Impact Assessments (DPIA), although this is not a requirement for all projects. Municipalities also use other several other assessments, including the Data Ethics Decision Aid (DEDA), Artificial Intelligence Impact Assessment (AIIA), Impact Assessment for Algorithms and Human Rights (IAMA), or a Privacy Impact Assessment (PIA). The police’s aforementioned Big Data Quality Framework has been worked into a digital tool to make working with this framework easier and more viable.

Further, we find that transparency also plays a large role in translating paper to practice. There seems to be a common understanding that no algorithmic system is perfect. This is perceived as unproblematic, so long as the data professional is open and transparent about it, both within the own organization as in communication to citizens. Municipalities realize such transparency, e.g., through algorithm registries, standard contracts for procurement of algorithms. For the police, most notable is a very practical XAI toolkit that is currently under development. This toolkit aims to aid data professionals in being transparent about algorithmic limitations and to document their own decision-making processes and rationale. This toolkit was explicitly based on the Big Data Quality Framework and as such can be seen as another translation of this particular document to a practical tool.

In conclusion, in order to cope with the translation of paper to practice, municipalities and the police create and implement tools that fit more closely to their daily practice. This shows there is an intention and perceived responsibility to find ways to implement rules, both those anchored in laws and those that are not. However, this translation from paper to tool is still a relatively new and ongoing process and not yet applicable to all situations. As a data protection officer at a municipality notes: “The Association of Dutch Municipalities (VNG) has a DPIA tool, for example. It’s a kind of questionnaire that you work through and helps identifies risks and mitigation strategies. Great, a DPIA tool! But Jesus Christ, after an hour I am absolutely done, except you are nowhere near done! It needs to be practical. So I said: ‘it’s great, but I won’t use it’” (T1).

Translating the paper framework to a practical tool thus does not necessarily ensure that the gap between paper and the daily practice of a data professional is sufficiently bridged. The paper framework needs to be practically fleshed out, through tools like impact assessments or deliberation tools. Yet these tools, again, need to be tailored to practice. The everyday practice of dealing with ethical and legal frameworks is, put simply, quite situated (cf. Kuiper^34^).

Data professionals at both municipalities and the police report that, despite many laws, guidelines, and frameworks existing, they mostly have to rely on their own education and expertise. As noted by one participant in the police: “You try to estimate what is desirable and what isn’t (…) rather than basing it on policy or rules” (Michel). Although this works for some of the larger municipalities and the police, some of the smaller municipalities lack such expertise or capacity. Further, knowledge is often situated with a small group within an organization, risking giving this small group a great deal of control to the small group responsible for the translation process.

### Reflection on the use of frameworks

Using the analytical research framework developed in Analytical research framework as an analytical lens for our own data allowed us to experience firsthand the problems data professionals might encounter when working with frameworks in practice. This is particularly true as our data are very practice oriented. In this section, we reflect critically on our own experience as researchers in using the framework, and what we have learned by using this method. We found the research framework helped us solidify our thinking about the various dimensions mentioned in it. By making our thinking about each point explicit, we were able to recognize differences and similarities between our research sites. This enabled us to find interesting details in our data that we might have otherwise missed or considered irrelevant or obvious. As such, the framework was a very useful point of departure in structuring, organizing, and analyzing our heterogeneous material. We quickly found that by just filling each element of the framework, we lacked shared language and had different interpretations of the points in the framework. Applying the framework to our data thus required an extensive dialog amongst us as researchers.

One key issue we encountered is the fact that a framework exists of ten separate points. Practice is much fuzzier than such a framework seems to suggest. As a result, many of the points overlapped greatly, and separating them at times felt artificial. For example, while they are clearly separated in our framework, in practice there is much overlap between preventing misuse and ensuring privacy and security. Both these issues may be safeguarded, for instance, through data security measures. As such measures might further be codified in laws, such as laws defining who should have access to which data, a further overlap emerges with the lawful basis portion of the framework. Separating the analytical discussion of these points without repeating ourselves thus proved a challenge. This was further strengthened by the nature of our data: as our data were very practice oriented, it was difficult to fit it into the separate categories of this framework.

Although we did find it a useful starting point for our discussion, it also left us at times unsatisfied and frustrated in ways we initially did not foresee. Using a framework requires a lot of operationalization on the side of the researcher or data professional trying to use it. In our case, this led us to rewriting the empirical section of this paper multiple times. We took numerous steps to move back and forth between our analytical framework and the empirical data, trying to ensure that categories fit correctly and that they accurately represented the data, while acknowledging where the fit was not perfect.

Thus, working with our own analytical research framework has given us a more in-depth understanding of the issues a data professional might encounter in trying to implement legal and ethical frameworks in their daily practice. Although frameworks do certainly have a role in data science and they can form a good starting point in governing data science practices in the public sector, it is essential to be aware of their limitations.

Working with a legal or ethical framework requires a lot of time, discussion, and operationalization on the part of the data professional. It requires the data professionals to establish clear interpretations and shared language. If this is not made explicit, and if these shared interpretations do not exist on an organizational level, this might lead to confusion in the application of the framework. Even when such clear interpretations exist, they might not apply to the practical situation at hand directly. Due to their distance from practical realities and their abstract nature, frameworks cannot function as a fix-all to ensure responsible data science practices. Operationalization’s more closely related to the daily practice of data professionals are necessary to govern data science practices in municipalities and the Netherlands police.

### Limitations

There are some important limitations to our research. First of all, our research is limited to everyday practices of individuals and cannot be considered a sufficient reflection of algorithmic infrastructural systems, nor is sufficient information on these infrastructures available yet. This is partially because, at an infrastructural level, there are not often regular audits on these applications*,* although there is an increasing demand for them at numerous different levels.^48^ While, for instance, the Dutch Court of Audit released an assessment framework for algorithms, and the Dutch Government announced a new algorithm watchdog, the infrastructural data that these audits could provide are currently very sparse. Thus, we are for example unable to observe the ways in which contractual relationships with specific technology providers influence the ways in which data professionals operate. These broader infrastructural dimensions are beyond the scope of this paper, which is very much focused on everyday practices and the lived professional experiences of individuals.

As we have only collected data in the Netherlands, the scope of our findings is also limited to the context of public sector organizations and the specific context of the Netherlands. Insofar as other countries or sectors share similar contexts, there may be similarities to studies conducted in these similar contexts. However, as the goal of this study was gaining a deeper understanding of the cases we were looking at, any attempts at generalization of our results beyond the Dutch public sector context should proceed with caution.

Finally, our findings are limited by the total number of interviews (24) and field notes (97) that were possible within our qualitative research, a significant part of which took place during the COVID-19 pandemic. Had we spoken to more or different interviewees, we might have reached different conclusions. This is a common limitation in qualitative research, but due to limitations in possible data collection related to a pandemic, we consider it important to emphasize it again here.

### Conclusions

How do ethical and legal frameworks influence the everyday practices on data and algorithms of public sector data professionals in the Netherlands? These ethical and legal frameworks clearly play a role in the practices of data professionals, but perhaps in a different way than we expected when we started the paper. There remains a considerable disconnect between the frameworks themselves and the actual everyday practices of individuals. This disconnect is often linked to practicability of the frameworks themselves, with individuals lacking the time and capacity to engage meaningfully with these frameworks.

Instead, key elements of the frameworks are integrated into professional practices implicitly, influencing the decision making of data professionals in less-well-documented ways. This mechanism of integration provides for lots of points in which numerous relevant human rights are meaningfully debated but for little accountability for this process in everyday practice. Decision making about responsible and accountable data practices is often delegated to a data protection officer, a legal professional, or a software tool, as many of the individuals involved typically do not feel competent or do not have the relevant skillset to make these decisions themselves.

What is also clear is the limited efficacy of tools and processes that are perceived to take too long or to be too complex or bureaucratic. The discretionary power of data professionals means that numerous ethical and legal frameworks often remain unused. Notably, the main value to improve these everyday practices is not to provide for more ethical frameworks or principles but rather in the creation of a vibrant public information climate^49^ that constantly raises and problematizes key questions and tensions within the framework. Public debates about technology ethics, legal rules, and human rights clearly influence the everyday practices of data professionals, even if this influence is embedded in diffuse professional practices.

Another key challenge is the tendency to attempt to resolve all dilemmas at the beginning of a project and then avoid them throughout the rest of the project life cycle. Instead, systematic evaluation through the whole project life cycle is far more effective and allows projects to adapt to new challenges and issues over time.

Notably, having studied and selected both of our cases with a most different case design, what we find very interesting is that challenges are very similar in both organizations, regardless of the degree of technical capacity within the organization of the organization’s mandate. Our analysis seems to suggest that the challenges we have found are problems that cut across both different disciplinary backgrounds and different organizational mandates.

Based on these findings, we believe that there is a need for wider data literacy and a deeper understanding of legal and ethical questions and trade-offs within the public sector. Data professionals currently have far too much autonomy and discretion because most of the actors they are talking to do not have a similar level of literacy. In this sense we agree with Møller, Shklovski and Hildebrandt, that data professionals “discretion [is] the main access point for human values to enter society’s decision-making practices.”^16^ There is also a need for additional capacity to ensure that data professionals have the time to consider legal and ethical questions more systematically.

Finally, one common thread we encountered toward outsourcing difficult questions to data protection officers or lawyers should be avoided. Instead, these difficult questions belong to the core of public sector operations and should be treated as such. Mainstreaming literacy, rather than specialization, is from our perspective a necessary consequence of situating these questions at the core of public sector operations.

What will not help, however, is yet another ethical framework or set of high-level principles. Instead, far greater effort needs to be put into operationalizing and systematizing existing legal and ethical questions into the everyday practices and processes in the public sector. This will not always be fast or easy, but in the long term, institutionalization of existing informal debates provides an opportunity to add additional layers or transparency and accountability that are currently lacking. Working out the interface between law and professional practices^40^ remains key to ensuring the effective implementation of ethical and legal frameworks. We hope that this paper can help contribute to a better understanding of how these frameworks are implemented in practice.

## Data Availability

This research project has generated interview transcripts and field notes. Due to legal confidentiality agreements that formed part of the research process and were a condition to receiving access to the research sites, the interview transcripts and field notes are not publicly available.
